# Alcohol consumption, cigarette smoking, and familial breast cancer risk: findings from the Prospective Family Study Cohort (ProF-SC)

**DOI:** 10.1186/s13058-019-1213-1

**Published:** 2019-11-28

**Authors:** Nur Zeinomar, Julia A. Knight, Jeanine M. Genkinger, Kelly-Anne Phillips, Mary B. Daly, Roger L. Milne, Gillian S. Dite, Rebecca D. Kehm, Yuyan Liao, Melissa C. Southey, Wendy K. Chung, Graham G. Giles, Sue-Anne McLachlan, Michael L. Friedlander, Prue C. Weideman, Gord Glendon, Stephanie Nesci, kConFab Investigators, Irene L. Andrulis, Saundra S. Buys, Esther M. John, Robert J. MacInnis, John L. Hopper, Mary Beth Terry

**Affiliations:** 1 Department of Epidemiology, Mailman School of Public Health, Columbia University, 722 W. 168th Street, Room 1611, New York, NY 10032 USA; 2 Lunenfeld-Tanenbaum Research Institute, Sinai Health System, Toronto, Ontario Canada; 3 Dalla Lana School of Public Health, University of Toronto, Toronto, Ontario Canada; 4 Herbert Irving Comprehensive Cancer Center, Columbia University Irving Medical Center, New York, NY USA; 5 Centre for Epidemiology and Biostatistics, The University of Melbourne, Parkville, Victoria Australia; 6 Department of Medical Oncology, Peter MacCallum Cancer Centre, Melbourne, Victoria Australia; 7 Sir Peter MacCallum Department of Oncology, The University of Melbourne, Melbourne, Victoria Australia; 8 Department of Clinical Genetics, Fox Chase Cancer Center, Philadelphia, PA USA; 9 Cancer Epidemiology Division, Cancer Council Victoria, Melbourne, Victoria Australia; 10 Precision Medicine, School of Clinical Sciences at Monash Health, Monash University, Clayton, Victoria Australia; 11 Department of Clinical Pathology, The University of Melbourne, Parkville, Victoria Australia; 12 Departments of Pediatrics and Medicine, Columbia University, New York, NY USA; 13 Department of Medicine, St Vincent’s Hospital, The University of Melbourne, Parkville, Victoria Australia; 14 Department of Medical Oncology, St Vincent’s Hospital, Fitzroy, Victoria Australia; 15 Prince of Wales Clinical School, University of New South Wales, Sydney, New South Wales Australia; 16 Department of Medical Oncology, Prince of Wales Hospital, Randwick, New South Wales Australia; 17 The Research Department, The Peter MacCallum Cancer Centre, Melbourne, Victoria Australia; 18 Departments of Molecular Genetics and Laboratory Medicine and Pathobiology, University of Toronto, Toronto, Ontario Canada; 19 Department of Medicine and Huntsman Cancer Institute, University of Utah Health Sciences Center, Salt Lake City, UT USA; 20 Department of Medicine and Stanford Cancer Institute, Stanford University School of Medicine, Stanford, CA USA

**Keywords:** Breast cancer, Alcohol consumption, Cigarette smoking, Familial risk, BOADICEA

## Abstract

**Background:**

Alcohol consumption and cigarette smoking are associated with an increased risk of breast cancer (BC), but it is unclear whether these associations vary by a woman’s familial BC risk.

**Methods:**

Using the Prospective Family Study Cohort, we evaluated associations between alcohol consumption, cigarette smoking, and BC risk. We used multivariable Cox proportional hazard models to estimate hazard ratios (HR) and 95% confidence intervals (CI). We examined whether associations were modified by familial risk profile (FRP), defined as the 1-year incidence of BC predicted by Breast Ovarian Analysis of Disease Incidence and Carrier Estimation Algorithm (BOADICEA), a pedigree-based algorithm.

**Results:**

We observed 1009 incident BC cases in 17,435 women during a median follow-up of 10.4 years. We found no overall association of smoking or alcohol consumption with BC risk (current smokers compared with never smokers HR 1.02, 95% CI 0.85–1.23; consuming ≥ 7 drinks/week compared with non-regular drinkers HR 1.10, 95% CI 0.92–1.32), but we did observe differences in associations based on FRP and by estrogen receptor (ER) status. Women with lower FRP had an increased risk of ER-positive BC associated with consuming ≥ 7 drinks/week (compared to non-regular drinkers), whereas there was no association for women with higher FRP. For example, women at the 10th percentile of FRP (5-year BOADICEA = 0.15%) had an estimated HR of 1.46 (95% CI 1.07–1.99), whereas there was no association for women at the 90th percentile (5-year BOADICEA = 4.2%) (HR 1.07, 95% CI 0.80–1.44). While the associations with smoking were not modified by FRP, we observed a positive multiplicative interaction by FRP (*p*_interaction_ = 0.01) for smoking status in women who also consumed alcohol, but not in women who were non-regular drinkers.

**Conclusions:**

Moderate alcohol intake was associated with increased BC risk, particularly for women with ER-positive BC, but only for those at lower predicted familial BC risk (5-year BOADICEA < 1.25). For women with a high FRP (5-year BOADICEA ≥ 6.5%) who also consumed alcohol, being a current smoker was associated with increased BC risk.

## Background

Alcohol consumption is an established breast cancer (BC) risk factor, with a 7–10% increase in BC risk for each standard alcoholic drink consumed daily (12 fl oz. of beer, 5 fl oz. of wine, or 1.5 fl oz. of hard liquor) [1–6]. The association between cigarette smoking and BC risk has been less consistently observed, but the current weight of evidence points to a modest association [7–9]. A recent meta-analysis of 49 epidemiologic studies reported an 11% increase in BC risk for current smokers (RR 1.11, 95% CI 1.06–1.16), compared with never smokers [9]. Since alcohol is an established and modest risk factor for BC, concerns of residual confounding by alcohol consumption when examining the association of smoking with BC risk have been raised [2]. Some evidence points more to synergy between the two risk factors. For example, in a pooled analysis of 14 cohorts that stratified by amount of alcohol intake, the elevated BC risk associated with being a current smoker compared with non-current smokers was only observed for women who consumed alcohol [8].

The majority of epidemiological evidence has been collected from women at general population risk and has included few high-risk women; few studies have examined these modifiable factors for women across the spectrum of absolute BC risk. In the limited number of prospective studies with adequate sample size, no clear association between alcohol consumption and BC risk has been observed in *BRCA1* or *BRCA2* mutation carriers (for review see [10]). A recent prospective study of mutation carriers reported weak evidence of increased BC risk for current smokers compared with never smokers (HR 1.28; 95% CI 1.00–1.64) [11].

Estimates of relative risks from cohorts with large variation in absolute BC risk are important to understand associations with modifiable factors on an absolute risk scale [12]. For example, we have found that several risk factors for BC do not vary by FRP (tested as multiplicative interactions) including benign breast disease and high body mass index (BMI), which is important to highlight because it means that on an absolute level, women at higher BC risk may benefit more from the relative risk reductions [13–16]. Of the few studies that have examined interaction between BC family history and either alcohol consumption or cigarette smoking, three have suggested similar associations regardless of family history [17–19], while others found a lack of associations with smoking or alcohol for women with primarily a first-degree BC family history [4, 20–22]. These studies, however, were limited in evaluating interaction with BC family history, as only a small proportion (4–13%) of participants had a family history [18, 19, 21]. Additionally, most studies only examined family history as a binary construct (yes/no) and did not examine modification of risk across the spectrum of absolute BC risk. Thus, evidence from prospective cohorts with sufficient statistical power to examine the association of modifiable exposures across the spectrum is essential and an important first step in developing appropriate clinical recommendations for women across the risk spectrum, and particularly for women at the higher end of the risk spectrum who may only be given clinical advice on modifiable factors based on average risk women and/or not advised at all. We examined whether cigarette smoking and alcohol consumption were associated with BC risk in women across the spectrum of absolute familial risk, using a prospective cohort enriched for women at familial or genetic risk.

## Methods

### Study population

The Prospective Family Study Cohort (ProF-SC) [12] includes women enrolled in the Breast Cancer Family Registry (BCFR) which includes six breast cancer family studies in the USA, Canada, and Australia [23], and the Kathleen Cuningham Foundation Consortium for research into Familial Breast cancer (kConFab) Follow-up Project [24, 25]. All probands and their family members were followed prospectively from baseline for cancer and other health outcomes [12]. Screening for germline *BRCA1* and *BRCA2* mutations was conducted, as previously described [23, 26, 27]. The institutional review board at each participating study center approved the BCFR and kConFab, and all participants provided written informed consent.

In the current analysis, we studied women unaffected with breast cancer, aged 18 to 79 years at recruitment (baseline), who had at least 2 months of follow-up, and did not to have a bilateral risk-reducing mastectomy at baseline (*N* = 17,780). We excluded 191 women without sufficient pedigree data to allow calculation of a lifetime BC risk score using the Breast Ovarian Analysis of Disease Incidence and Carrier Estimation Algorithm (BOADICEA), and 236 women for whom we did not have complete data on alcohol consumption (whether they were regular or non-regular drinkers, as defined below) and cigarette smoking (whether they were current, former, or never smokers). From the 17,780 unaffected women in the original cohort, this left 17,435 (98.1%) available for analysis.

### Questionnaires

The BCFR and kConFab used the same core questionnaires at baseline [12]. The questionnaires asked about the participants’ demographic characteristics; education; race/ethnicity; height and weight; menstrual and reproductive history, including age at menarche, parity, breastfeeding, age at first birth, and age at menopause; hormonal birth control; menopausal hormone therapy use; medical history including diagnosis of breast or ovarian cancer, and breast or ovarian surgeries; and behavioral factors including cigarette smoking and alcohol consumption. Probands also completed a family history questionnaire that asked about breast and other cancers in their first-degree and second-degree relatives. Each participant’s cancer information was obtained from one or more sources and was usually self-reported or reported by a first-degree relative. We confirmed reported invasive BC diagnosis through pathology reports or cancer registry linkages for 81% of incident cases.

### Definitions of cigarette smoking and alcohol consumption

Baseline questionnaires included a detailed assessment of lifetime cigarette smoking and alcohol consumption, including questions about current and former smoking and drinking, duration of smoking and drinking, age at smoking and drinking initiation, average numbers of cigarettes smoked per day, and average number of each type of alcoholic drink (beer, wine/wine cooler, and liquor) consumed per week.

We classified women as ever smokers if they answered “yes” and as never smokers if they answered “no” to the following question: “Have you smoked at least one cigarette per day for 3 months or longer?”. For ever smokers, we defined additional exposure variables, including smoking status (former or current), age at smoking initiation (< 16, 16 to < 18, 18 to < 20, or ≥ 20 years), smoking duration (< 10, 10 to < 20, 20 to < 30, or ≥ 30 years), and smoking intensity (1 to < 5, 5 to < 10, 10 to < 20, or ≥ 20 cigarettes per day). We classified women as regular drinkers if they answered “yes” and as non-regular drinkers if they answered “no” to the following question: “Have you ever consumed any alcoholic beverages, such as beer, wine, or liquor, at least once a week for 6 months or longer?”. For regular drinkers, we defined additional exposure variables, including age at drinking initiation (< 18, 18 to < 21, 21 to < 30, or ≥ 30 years) and number of alcoholic drinks consumed per week (< 7 or ≥ 7), a common cut point in the literature. We defined one drink as a 12 oz. serving of beer, one medium glass of wine or wine cooler, or one shot of liquor, and calculated alcoholic drinks per week as the sum of the intake of each of the three different types of alcoholic beverages consumed.

### Familial risk profile

For each participant, we calculated a 1-year, 10-year, and lifetime (from birth to age 80 years) risk of invasive BC from multigenerational pedigree data on breast and ovarian cancer in relatives using the BOADICEA version 3 [28, 29]. This algorithm uses information on ovarian and female and male breast cancer and age at diagnosis for first-, second-, and third-degree relatives (where available), along with date of birth, vital status, age at interview or death, and country- and age-specific breast cancer incidence to calculate risk. Where available, information on *BRCA1* and *BRCA2* mutation testing was also used to calculate risk. We hereafter refer to this continuous risk score as the familial risk profile (FRP). A previous validation study of family cancer history information communicated within families in the BCFR found high sensitivity and specificity for family history of breast cancer [30]. Additionally, a recent validation study of commonly used breast cancer risk prediction models in ProF-SC found BOADICEA to be well calibrated (ratio of expected cases to observed cases 1.05 (95% CI 0.97–1.14); C-statistic 0.70 (95% CI 0.68–0.72)) [31].

### Statistical methods

We used Cox proportional hazard regression models with age as the time scale to estimate hazard ratios (HR) and their 95% confidence intervals (CI) for BC associated with FRP and each smoking and alcohol variable. We calculated person-years from 2 months after the age at completion of the baseline questionnaire to the age at diagnosis of BC or the earliest of the following events: age at risk-reducing mastectomy, age at death, age 80 years, or age last known to be alive. We used a robust variance estimator to account for the family structure of the cohort. We incorporated left-truncation in all models to avoid potential survivor bias. All models were stratified by birth cohort (< 1950, 1950–1959, 1960–1969, ≥ 1970) and adjusted for race/ethnicity (non-Hispanic white; non-Hispanic black; Hispanic; Asian; other) and study center. We considered the following variables measured at baseline as potential confounders: age at baseline (continuous), body mass index (BMI, continuous), education (≤ high school or general education development; vocational, technical, some college, or some university; bachelor or graduate degree), age at menarche (continuous), parity and breastfeeding (nulliparous; 1–2 full-term pregnancies and did not breast feed; 1–2 full-term pregnancies and breastfed; ≥ 3 full-term pregnancies and did not breast feed; ≥ 3 full-term pregnancies and breastfed), age at first birth (continuous and centered at mean), oral contraceptive use (current, former, never user), menopausal hormone use (current, former, never user), and menopausal status (pre- or post-menopausal). We also assessed for confounding by alcohol consumption (regular or non-regular drinker) in the smoking models and confounding by cigarette smoking (current, former, and never smoker) in the alcohol models. We included as confounding variables in the final models any variable that changed the smoking or drinking parameter estimate of interest by more than 10%. We assessed multiplicative interaction with FRP using the 1-year BOADICEA risk score and each smoking and alcohol variable of interest by including a cross-product term in the model and assessing the corresponding beta coefficient using the Wald test. We also estimated associations by estrogen receptor (ER) status (positive or negative), where the alternative ER subtype was censored at diagnosis. For example, ER-positive cases were censored at diagnosis in models examining ER-negative breast cancer. For ease of interpretation, we also present HRs by high and low FRP, using 0.34% as a categorical cutoff for absolute 1-year risk because it is the 1-year equivalent to the 5-year risk cutoff of 1.67%. We performed the following sensitivity analyses: including only those with confirmed invasive BC based on pathology reports or cancer registry linkages (81% of all cases were confirmed invasive), where unconfirmed cancers were censored at diagnosis, excluding those with a prior diagnosis of any cancer (except non-melanoma skin cancer) at baseline, and excluding *BRCA1* and *BRCA2* mutation carriers. We assessed the proportional hazards assumption by evaluating the Schoenfeld residuals. All statistical tests were two sided, and *p* values < 0.05 were considered statistically significant. All statistical analyses were performed using SAS software version 9.4 (SAS Institute Inc., Cary, NC, USA).

## Results

We followed 17,435 women from 6948 families in the BCFR and kConFab with an average age at enrollment into the cohorts of 46.7 years. During the 181,062 person-years of follow-up (median 10.4, maximum 24.0 years), there were 1009 incident cases of BC with an average age at diagnosis of 56.2 years. Of the 17,435 women, 15% (*n* = 2602) reported currently smoking at baseline, 27% (*n* = 4675) reported formerly smoking, and 58% (*n* = 10,158) reported being never smokers. Current smokers smoked more intensely (mean cigarettes/day 15.1 vs 13.1) and for a longer period (mean duration 23.9 vs 14.5 years) than former smokers (Table 1). Overall, 49% (*n* = 8618) of women reported being regular drinkers of alcoholic beverages at baseline and reported consuming an average of 7.7 total alcoholic drinks/week. Compared with never smokers, current and former smokers were more likely to be regular drinkers (63% and 66%, respectively, vs 38% for never smokers) and consumed more alcoholic drinks per week (mean drinks per week 10.9 and 8.0, respectively, compared with 6.0 for never smokers).

**Table 1:** Descriptive characteristics of women in the Breast Cancer Prospective Family Study Cohort (ProF-SC) by smoking status and alcohol consumption

	Non-smoker (*N* = 10,158)	Former smoker (*N* = 4675)	Current smoker (*N* = 2602)	Non-regular drinker (*N* = 8817)	Regular drinker (*N* = 8618)	Total cohort (*N* = 17,435)
Age at baseline (mean, SD)	46.5 ± 15.6	49.2 ± 14.2	43.6 ± 14.0	48.0 ± 15.7	45.5 ± 14.3	46.8 ± 15.1
Age at breast cancer diagnosis (mean, SD)	55.9 ± 12.2	58.2 ± 12.3	52.9 ± 11.5	57.0 ± 12.2	55.5 ± 12.3	56.2 ± 12.3
BOADICEA full lifetime risk score, % (mean, SD)	23.8 ± 17.2	22.7 ± 15.4	23.3 ± 16.3	23.6 ± 17.1	23.2 ± 16.0	23.4 ± 16.6
BOADICEA 1-year risk score, % (mean, SD)	0.5 ± 0.6	0.5 ± 0.6	0.4 ± 0.6	0.5 ± 0.7	0.4 ± 0.6	0.5 ± 0.6
BOADICEA 5-year risk score, % (mean, SD)	2.4 ± 3.1	2.6 ± 3.1	2.2 ± 2.8	2.5 ± 3.2	2.4 ± 2.9	2.4 ± 3.0
Body mass index, kg/m^2^ (mean, SD)	25.8 ± 5.6	26.3 ± 5.7	25.3 ± 5.7	26.5 ± 5.9	25.2 ± 5.3	25.8 ± 5.7
Cigarette smoking variables						
Age at smoking initiation, years (mean, SD)	NA	18.7 ± 5.2	18.1 ± 5.4	18.9 ± 6.0	18.3 ± 4.8	18.5 ± 5.3
Smoking duration, years smoked (mean, SD)	NA	14.5 ± 11.6	23.9 ± 13.4	19.0 ± 13.6	17.0 ± 12.7	17.7 ± 13.1
Smoking intensity, cigarettes/day (mean, SD)	NA	13.1 ± 10.8	15.1 ± 9.3	14.0 ± 10.6	13.7 ± 10.1	13.8 ± 10.3
Smoking intensity, cigarettes/day (*n*, %)						
Non-smoker^a^	10,158 (100.0)	NA	NA	6286 (71.3)	3872 (44.9)	10,158 (58.3)
1 to < 5 cig/day	NA	992 (21.2)	242 (9.3)	413 (4.7)	821 (9.5)	1234 (7.1)
5 to < 10 cig/day	NA	888 (19.0)	421 (16.2)	440 (5.0)	869 (10.1)	1309 (7.5)
10 to < 20 cig/day	NA	1321 (28.3)	936 (36.0)	823 (9.3)	1434 (16.6)	2257 (13.0)
≥ 20 more cig/day	NA	1434 (30.7)	980 (37.7)	830 (9.4)	1584 (18.4)	2414 (13.9)
Missing	0	40 (0.9)	23 (0.9)	25 (0.3)	38 (0.4)	63 (0.4)
Smoking duration, years smoked (*n*, %)						
Non-smoker	10,158 (100.0)	NA	NA	6286 (71.3)	3872 (44.9)	10,158 (58.3)
< 10 years	NA	1900 (40.6)	388 (14.9)	728 (8.3)	1560 (18.1)	2288 (13.1)
10 to < 20 years	NA	1367 (29.2)	582 (22.4)	621 (7.0)	1328 (15.4)	1949 (11.2)
20 to < 30 years	NA	701 (15.0)	646 (24.8)	507 (5.8)	840 (9.8)	1347 (7.7)
≥ 30 years	NA	622 (13.3)	821 (31.6)	585 (6.6)	858 (10.0)	1443 (8.3)
Missing	0	85 (1.8)	165 (6.3)	90 (1.0)	160 (1.9)	250 (1.4)
Alcohol consumption variables						
Total drinks per week (mean, SD)	6.0 ± 7.0	8.0 ± 10.1	10.9 ± 14.0	NA	7.7 ± 10.0	7.7 ± 10.0
Age at drinking initiation (mean, SD)	25.5 ± 11.0	23.7 ± 9.8	22.8 ± 8.6	NA	24.4 ± 10.2	24.4 ± 10.2
Drinking duration, years (mean, SD)	15.2 ± 12.0	18.8 ± 12.9	16.0 ± 11.8	NA	16.6 ± 12.4	16.6 ± 12.4
Categorical drinks per week (*n*, %)						
Non-regular drinker^c^	6406 (63.1)	1686 (36.1)	986 (37.9)	8817 (100%)	261 (3.0)^c^	9078 (52.1)
< 7 drinks/week	2420 (23.8)	1654 (35.4)	763 (29.3)	NA	4837 (56.1)	4837 (27.7)
≥ 7 drinks/week	1095 (10.8)	1158 (24.8)	725 (27.9)	NA	2978 (34.6)	2978 (17.1)
Missing	237 (2.3)	177 (3.8)	128 (4.9)	NA	542 (6.3)	542 (3.1)
*BRCA* mutation carriers						
Non-carrier	9374 (92.3)	4405 (94.2)	2430 (93.4)	8148 (92.4)	8061 (93.5)	16,209 (93.0)
*BRCA1* carrier	417 (4.1)	156 (3.3)	105 (4.0)	366 (4.2)	312 (3.6)	678 (3.9)
*BRCA2* carrier	367 (3.6)	114 (2.4)	67 (2.6)	303 (3.4)	245 (2.8)	548 (3.1)
Study center						
Philadelphia	444 (4.4)	246 (5.3)	94 (3.6)	420 (4.8)	364 (4.2)	784 (4.5)
New York	1211 (11.9)	616 (13.2)	171 (6.6)	1074 (12.2)	924 (10.7)	1998 (11.5)
Utah	666 (6.6)	64 (1.4)	38 (1.5)	617 (7.0)	151 (1.8)	768 (4.4)
Australia	2147 (21.1)	1046 (22.4)	725 (27.9)	1864 (21.1)	2054 (23.8)	3918 (22.5)
Ontario, Canada	1241 (12.2)	703 (15.0)	418 (16.1)	1160 (13.2)	1202 (14.0)	2362 (13.6)
Northern California	1892 (18.6)	679 (14.5)	380 (14.6)	1924 (21.8)	1027 (11.9)	2951 (16.9)
kConFab	2557 (25.2)	1321 (28.3)	776 (29.8)	1758 (19.9)	2896 (33.6)	4654 (26.7)
Birth cohort						
< 1950	3917 (38.6)	2108 (45.1)	797 (30.6)	3795 (43.0)	3027 (35.1)	6822 (39.1)
1950–1959	2291 (22.6)	1151 (24.6)	666 (25.6)	1975 (22.4)	2133 (24.8)	4108 (23.6)
1960–1969	2002 (19.7)	904 (19.3)	638 (24.5)	1590 (18.0)	1954 (22.7)	3544 (20.3)
≥ 1970	1948 (19.2)	512 (11.0)	501 (19.3)	1457 (16.5)	1504 (17.5)	2961 (17.0)
Race						
Non-Hispanic White	7803 (76.8)	3998 (85.5)	2158 (82.9)	6397 (72.6)	7562 (87.8)	13,959 (80.1)
Non-Hispanic Black	481 (4.7)	170 (3.6)	158 (6.1)	499 (5.7)	310 (3.6)	809 (4.6)
Hispanic	1012 (10.0)	287 (6.1)	137 (5.3)	1057 (12.0)	379 (4.4)	1436 (8.2)
Asian	569 (5.6)	64 (1.4)	32 (1.2)	545 (6.2)	120 (1.4)	665 (3.8)
Other	244 (2.4)	135 (2.9)	94 (3.6)	271 (3.1)	202 (2.3)	473 (2.7)
Missing	49 (0.5)	21 (0.5)	23 (0.9)	48 (0.5)	45 (0.5)	93 (0.5)
Education						
High school graduate or less	3161 (18.1)	1515 (8.7)	1139 (6.5)	3391 (38.5)	2424 (28.1)	5815 (33.4)
Some college/vocational school	3656 (21.0)	1838 (10.5)	1106 (6.3)	3166 (35.9)	3434 (39.9)	6600 (37.9)
Bachelor’s/graduate degree	3296 (18.9)	1311 (7.5)	342 (2.0)	2213 (25.1)	2736 (31.8)	4949 (28.4)
Missing	45 (0.3)	11 (0.1)	15 (0.1)	47 (0.5)	24 (0.3)	71 (0.4)
Parity (number of full-term pregnancies/breastfeeding history)						
Nulliparous	2617 (15.0)	871 (5.0)	660 (3.8)	1857 (21.1)	2291 (26.6)	4148 (23.8)
Parous 1–2/no breastfeeding	1037 (6.0)	586 (3.4)	410 (2.4)	1140 (12.9)	893 (10.4)	2033 (11.7)
Parous 1–2/breastfed	2626 (15.1)	1395 (8.0)	667 (3.8)	2085 (23.7)	2603 (30.2)	4688 (26.9)
Parous 3+/no breastfeeding	758 (4.4)	390 (2.2)	256 (1.5)	854 (9.7)	550 (6.4)	1404 (8.1)
Parous 3+/breastfed	3047 (17.5)	1387 (8.0)	591 (3.4)	2820 (32.0)	2205 (25.6)	5025 (28.8)
Missing	73 (0.4)	46 (0.3)	18 (0.1)	61 (0.7)	76 (0.9)	137 (0.8)
Hormonal birth control us						
Never	2832 (16.2)	933 (5.4)	469 (2.7)	2756 (31.3)	1478 (17.2)	4234 (24.3)
Former	5825 (33.4)	3194 (18.3)	1722 (9.9)	5017 (56.9)	5724 (66.4)	10,741 (61.6)
Current	1424 (8.2)	513 (2.9)	393 (2.3)	970 (11.0)	1360 (15.8)	2330 (13.4)
Missing	77 (0.4)	35 (0.2)	18 (0.1)	74 (0.8)	56 (0.7)	130 (0.8)

*NA* not applicable

^a^Smokers are defined as having smoked at least once cigarette per day for 3 months or longer

^b^Regular drinkers are defined as consuming one alcoholic beverage at least once a week for 6 months or longer

^c^This includes women who reported being regular drinkers that consumed 0 drinks per week. These women were categorized as non-regular drinkers in the categorical drinks per week variable

**Fig. 1:**
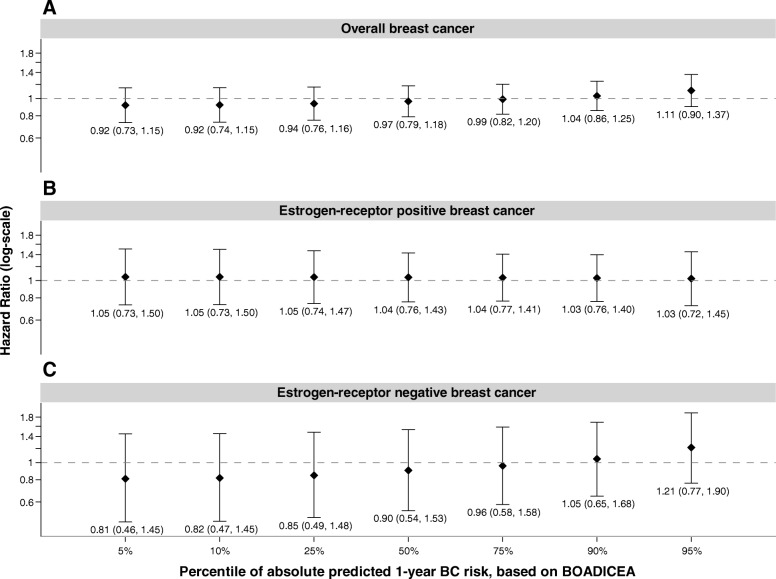
Associations of current smoking compared to never smoking and breast cancer (BC) risk by percentiles of absolute predicted 1-year BC risk for overall BC, estrogen receptor-positive BC, and estrogen receptor-negative BC. Hazard ratios (HR) reflect associations for current smokers compared to never smokers and breast cancer risk by percentiles of absolute predicted 1-year breast cancer risk, as estimated by BOADICEA  for overall breast cancer (panel **a**), estrogen-receptor positive breast cancer (panel **b**), and estrogen-receptor negative breast cancer (panel **c**). HRs are stratified by birth cohort and are adjusted for race/ethnicity, study center, education, oral contraceptive use, and body mass index

Overall, there was no statistically significant association between smoking status and BC risk (former smokers HR 1.06, 95% CI 0.92–1.22; current smokers HR 1.02, 95% CI 0.85–1.23, compared with never smokers). We also observed no statistically significant associations between smoking status and risk of ER-positive BC (former smokers HR 0.97, 95% CI 0.77–1.21; current smokers HR 1.04, 95% CI 0.77–1.4, compared with never smokers) or risk of ER-negative BC (former smokers HR 0.95, 95% CI 0.63–1.41; current smokers HR 1.22, 95% CI 0.78–1.91, compared with never smokers) (Table 2). Figure 1 illustrates the association of being a current smoker (as compared to never smoking) by percentiles of absolute predicted 1-year BC risk. Although the overall interaction term was statistically significant (*p* value 0.03), the individual HRs at different percentiles of FRP were not, with HRs of 1.04 (95% CI 0.86–1.25) and 0.92 (95% CI 0.74–1.15) for women in the 90th and 10th percentile of 1-year BOADICEA risk score, respectively (Fig. 1). These results were consistent for other measures of FRP we examined, including 10-year BOADICEA risk score and lifetime risk to age 80 (results not shown).

**Table 2:** Adjusted hazard ratios and 95% confidence intervals from Cox proportional hazards modeling of smoking status

		Overall BC			ER-positive BC			ER-negative BC		
Continuous FRP	Person years	BC	Model 1^a^	Model 2^b^	BC	Model 1^a^	Model 2^b^	BC	Model 1^a^	Model 2^b^
			HR (95% CI)	HR (95% CI)		HR (95% CI)	HR (95% CI)		HR (95% CI)	HR (95% CI)
Never smoker ^c^	102,781.5	559	Reference	Reference	235	Reference	Reference	85	Reference	Reference
Former smoker	47,088.8	302	1.06 (0.92, 1.22)	1.14 (0.96, 1.34)	118	0.97 (0.77, 1.21)	1.09 (0.84, 1.43)	39	0.95 (0.63, 1.41)	0.92 (0.59, 1.45)
Current smoker	26,466.7	137	1.02 (0.85, 1.23)	0.92 (0.73, 1.15)	54	1.04 (0.77, 1.40)	1.05 (0.73, 1.51)	27	1.22 (0.78, 1.91)	0.81 (0.46, 1.43)
Former smoker*FRP				0.91 (0.81, 1.02)			0.84 (0.68, 1.05)			1.02 (0.85, 1.22)
Current smoker*FRP				1.16 (0.99, 1.35)			0.99 (0.74, 1.32)			1.36 (1.08, 1.71)
Categorical FRP			1-year FRP < 0.34%	1-year FRP ≥ 0.34%		1-year FRP < 0.34%	1-year FRP ≥ 0.34%		1-year FRP < 0.34%	1-year FRP ≥ 0.34%
			HR (95% CI)	HR (95% CI)		HR (95% CI)	HR (95% CI)		HR (95% CI)	HR (95% CI)
Never smoker			Reference	Reference		Reference	Reference		Reference	Reference
Former smoker			1.11 (0.87, 1.43)	1.03 (0.87, 1.23)		0.97 (0.65, 1.45)	0.98 (0.74, 1.28)		0.80 (0.31, 2.04)	0.94 (0.61, 1.44)
Current smoker			0.91 (0.64, 1.28)	1.09 (0.87, 1.37)		0.90 (0.52, 1.57)	1.13 (0.79, 1.61)		0.87 (0.29, 2.67)	1.18 (0.72, 1.93)

*BC* breast cancer, *HR* hazard ratio, *CI* confidence interval, *ER* estrogen receptor expression, *FRP* familial risk profile

^a^Model 1 = Adjusted Cox models which are stratified by birth cohort (< 1950, 1950–1959, 1960–1969, ≥ 1970) and adjusted for study site, race/ethnicity, body mass index, education, and hormonal birth control use

^b^Model 2 = Interaction models that include an interaction term with FRP as estimated by 1-year BOADICEA risk score

^c^Smokers are defined as having smoked at least one cigarette per day for 3 months or longer

**Fig. 2:**
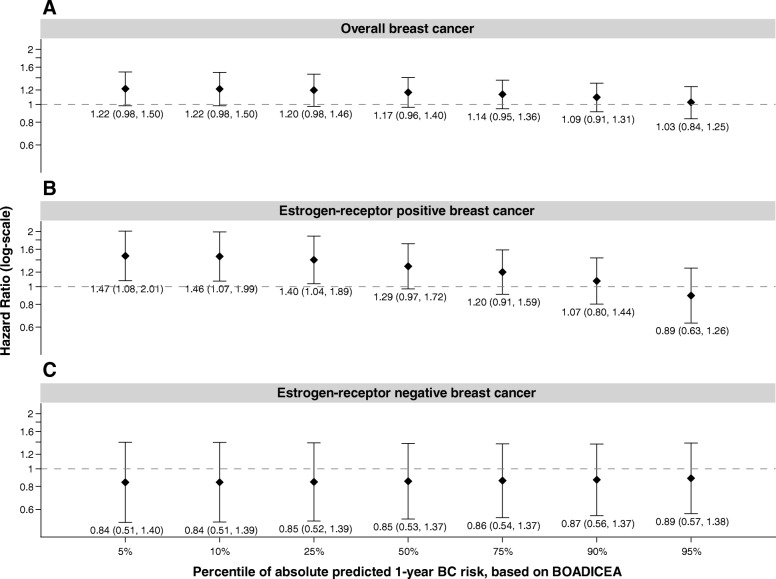
Associations of consuming ≥ 7 alcoholic drinks per week (compared to non-regular drinkers) and breast cancer risk by percentiles of absolute predicted 1-year breast cancer risk for overall breast cancer, estrogen receptor-positive breast cancer, and estrogen receptor-negative breast cancer. Hazard ratios (HR) reflect associations for consuming ≥ 7 alcoholic drinks per week compared to non-drinkers and breast cancer risk by percentiles of absolute predicted 1-year breast cancer risk, as estimated by BOADICEA for overall breast cancer (panel **a**), estrogen-receptor positive breast cancer (panel **b**), and estrogen-receptor negative breast cancer (panel **c**). HRs are stratified by birth cohort and are adjusted for race/ethnicity, study center, education, oral contraceptive use, body mass index, and cigarette smoking

Overall, there was no statistically significant association between alcohol consumption and overall BC (relative to non-regular drinkers, HR for < 7 drinks per week 0.99, 95% CI 0.85–1.16; HR for ≥ 7 drinks per week 1.10, 95% 0.92–1.32) (Table 3). We also observed no significant associations between alcohol consumption and risk of ER-positive BC (HR for < 7 drinks per week 1.07, 95% CI 0.84–1.36; HR for ≥ 7 drinks per week 1.15, 95% 0.87–1.51, compared with non-drinkers) or ER-negative BC (HR for < 7 drinks per week 0.69, 95% CI 0.46–1.04; HR for ≥ 7 drinks per week 0.89, 95% 0.57–1.39, compared with non-drinkers). While we found that FRP did not modify the association for overall BC (interaction *p* value 0.19), we did find differences by ER subtype. We observed a negative multiplicative interaction by FRP for higher alcohol intake (≥ 7 drinks/week) compared with non-regular drinkers for ER-positive BC. As illustrated in Fig. 2, women at the 10th (which translates to a 5-year BOADICEA of 0.15%) percentile of FRP had a 46% increased risk of ER-positive BC (HR 1.46, 95% CI 1.07–1.99), while there was no association for women in the 90th percentile (HR 1.07, 95% CI 0.80–1.44). We observed similar patterns when modeling alcohol use as a continuous variable (data not shown). These results were consistent for other measures of FRP we examined, including 10-year BOADICEA risk score and lifetime risk to age 80 (results not shown).

**Table 3:** Adjusted hazard ratios and 95% confidence intervals from Cox proportional hazards modeling of alcohol consumption

		Overall BC			ER-positive BC			ER-negative BC		
Continuous FRP	Person years	BC	Model 1^a^	Model 2^b^	BC	Model 1^a^	Model 2^b^	BC	Model 1^a^	Model 2^b^
			HR (95% CI)	HR (95% CI)		HR (95% CI)	HR (95% CI)		HR (95% CI)	HR (95% CI)
Non-regular drinker^c^	90,009.0	492	Reference	Reference	192	Reference	Reference	85	Reference	Reference*
< 7 drinks/week	48,947.2	275	0.99 (0.85, 1.16)	1.01 (0.84, 1.21)	122	1.07 (0.84, 1.36)	1.14 (0.85, 1.52)	36	0.69 (0.46, 1.04)	0.64 (0.40, 1.04)
≥ 7 drinks/week	29,982.8	193	1.10 (0.92, 1.32)	1.22 (0.99, 1.50)	80	1.15 (0.87, 1.51)	1.48 (1.08, 2.02)	30	0.89 (0.57, 1.39)	0.84 (0.51, 1.40)
< 7 drinks/week*FRP				0.99 (0.87, 1.12)			0.92 (0.74, 1.15)			1.06 (0.87, 1.29)
≥ 7 drinks/week*FRP				0.88 (0.76, 1.01)			0.68 (0.52, 0.89)			1.04 (0.85, 1.27)
Categorical FRP			1-year FRP < 0.34%	1-year FRP ≥ 0.34%		1-year FRP < 0.34%	1-year FRP ≥ 0.34%		1-year FRP < 0.34%	1-year FRP ≥ 0.34%
			HR (95% CI)	HR (95% CI)		HR (95% CI)	HR (95% CI)		HR (95% CI)	HR (95% CI)
Non-regular drinker			Reference	Reference		Reference	Reference		Reference	Reference
< 7 drinks/week			1.02 (0.77, 1.35)	0.96 (0.79, 1.16)		0.99 (0.64, 1.54)	1.10 (0.82, 1.48)		0.69 (0.26, 1.87)	0.65 (0.41, 1.03)
≥ 7 drinks/week			1.18 (0.87, 1.61)	1.05 (0.84, 1.31)		1.24 (0.76, 2.01)	1.09 (0.77, 1.53)		0.43 (0.13, 1.49)	0.98 (0.61, 1.58)

*BC* breast cancer, *HR* hazard ratio, *CI* confidence interval, *ER* estrogen receptor expression, *FRP* familial risk profile

^a^Model 1 = Adjusted Cox models which are stratified by birth cohort (< 1950, 1950–1959, 1960–1969, ≥ 1970) and adjusted for study site, race/ethnicity, body mass index, education, hormonal birth control use, and cigarette smoking

^b^Model 2 = Interaction models that include an interaction term with FRP as estimated by 1-year BOADICEA risk score

^c^Regular drinkers are defined as consuming one alcoholic beverage at least once a week for 6 months or longer

**Fig. 3:**
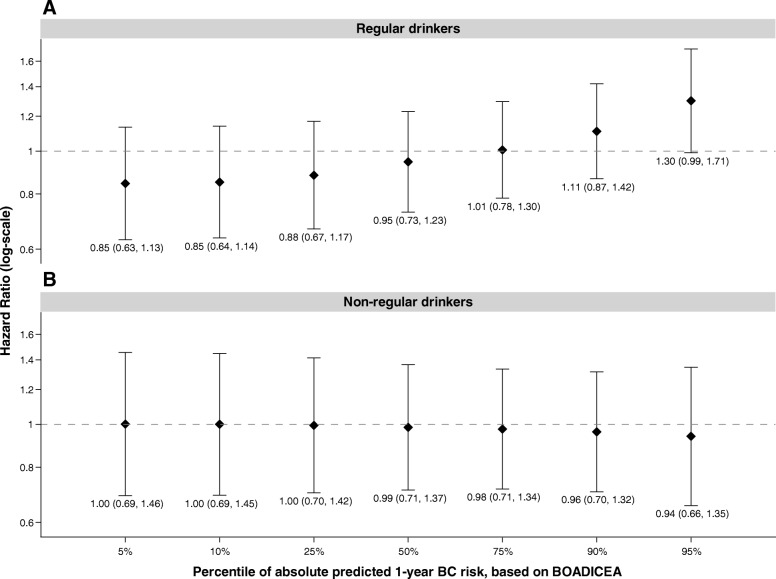
Associations of current smoking compared to never smoking and breast cancer risk by percentiles of absolute predicted 1-year breast cancer risk stratified by alcohol consumption at baseline. Hazard ratios (HR) reflect associations for current smokers compared to never smokers and breast cancer risk by percentiles of absolute predicted 1-year breast cancer risk, as estimated by BOADICEA, stratified by alcohol consumption status at baseline; regular drinkers are presented in panel **a** and non-regular drinkers in panel **b**. HRs are stratified by birth cohort and are adjusted for race/ethnicity, study center, education, oral contraceptive use, and body mass index

We found a significant three-way interaction for FRP, alcohol consumption (non-regular drinkers and regular drinkers), and cigarette smoking (current, former, never smokers) (interaction *p* value = 0.01). When we stratified models examining smoking and BC risk by alcohol consumption, we found no overall significant association for current or former smoking by regular and non-regular alcohol drinking (Table 4). We examined whether the association of smoking and BC is modified by FRP within strata of alcohol consumption and observed a statistically significant positive interaction with FRP for current vs never smokers for women who regularly consumed alcohol, but not for non-regular alcohol drinkers. While we found significant multiplicative interaction by FRP (*p* value = 0.005) for smoking status in women who also consumed alcohol, this was primarily driven by women with very high FRP (Fig. 3). As illustrated in Fig. 3, there was a 30% increased overall BC risk for women at the 95th percentile of FRP (5-year BOADICEA of 6.55%) who reported currently smoking at baseline and were regular drinkers (HR 1.30, 95% CI 0.99–1.71), and no association for current smokers with the same FRP, but were not regular drinkers (HR 0.94, 95% CI 0.66–1.35).

**Table 4:** Adjusted hazard ratios and 95% confidence intervals from Cox proportional hazards modeling of smoking status stratified by alcohol consumption

		Overall BC			ER-positive BC			ER-negative BC		
Continuous FRP	Person years	BC	Model 1^a^	Model 2^b^	BC	Model 1^a^	Model 2^b^	BC	Model 1^a^	Model 2^b^
			HR (95% CI)	HR (95% CI)		HR (95% CI)	HR (95% CI)		HR (95% CI)	HR (95% CI)
Regular drinkers^d^										
Never smoker	40,334.3	216	Reference	Reference	90	Reference	Reference	24	Reference	Reference
Former smoker	31,831.4	219	1.16 (0.96, 1.41)	1.23 (0.99, 1.53)	97	1.24 (0.93, 1.65)	1.25 (0.89, 1.75)	24	1.17 (0.64, 2.17)	1.08 (0.55, 2.12)
Current smoker	16,742.8	90	1.07 (0.83, 1.36)	0.84 (0.63, 1.13)	35	1.12 (0.76, 1.65)	0.99 (0.62, 1.60)	20	1.77 (0.91, 3.46)	0.95 (0.43, 2.07)
Former smoker*FRP				0.93 (0.78, 1.09)			0.99 (0.74, 1.34)			1.10 (0.83, 1.46)
Current smoker*FRP				1.39 (1.13, 1.71)			1.21 (0.80, 1.85)			1.67 (1.16, 2.40)
Non-regular drinkers										
Never smoker	62,284.47	343	Reference	Reference	145	Reference	Reference	61	Reference	Reference
Former smoker	15,157.38	83	0.88 (0.69, 1.12)	0.90 (0.67, 1.21)	21	0.54 (0.34, 0.86)	0.63 (0.36, 1.10)	15	0.95 (0.53, 1.72)	0.93 (0.45, 1.89)
Current smoker	9692.36	47	0.97 (0.71, 1.32)	1.00 (0.69, 1.46)	19	0.98 (0.61, 1.59)	1.04 (0.59, 1.84)	7	0.77 (0.34, 1.74)	0.68 (0.23, 1.97)
Former smoker*FRP^e^				0.98 (0.81, 1.17)			0.82 (0.54, 1.25)			1.02 (0.77, 1.35)
Current smoker*FRP^e^				0.95 (0.70, 1.29)			0.93 (0.61, 1.42)			1.11 (0.69, 1.79)
Categorical FRP			1-year FRP < 0.34%	1-year FRP ≥ 0.34%		1-year FRP < 0.34%	1-year FRP ≥ 0.34%		1-year FRP < 0.34%	1-year FRP ≥ 0.34%
			HR (95% CI)	HR (95% CI)		HR (95% CI)	HR (95% CI)		HR (95% CI)	HR (95% CI)
Regular drinkers										
Former smoker			1.16 (0.85, 1.59)	1.16 (0.92, 1.47)		1.04 (0.64, 1.69)	1.32 (0.93, 1.88)		0.60 (0.15, 2.44)	1.38 (0.71, 2.7)
Current smoker			0.81 (0.51, 1.27)	1.25 (0.94, 1.68)		0.87 (0.42, 1.78)	1.28 (0.80, 2.04)		0.70 (0.13, 3.63)	1.99 (0.98, 4.03)
Non-regular drinkers										
Former smoker			0.88 (0.54, 1.42)	0.89 (0.67, 1.18)		0.68 (0.28, 1.65)	0.52 (0.30, 0.90)		1.61 (0.45, 5.77)	0.83 (0.44, 1.57)
Current smoker			1.11 (0.65, 1.90)	0.91 (0.62, 1.35)		0.97 (0.39, 2.45)	1.06 (0.60, 1.88)		1.10 (0.22, 5.43)	0.66 (0.26, 1.68)

*BC* breast cancer, *HR* hazard ratio, *CI* confidence interval, *ER* estrogen receptor expression, *FRP* familial risk profile

^a^Model 1 = Adjusted Cox models which are stratified by birth cohort (< 1950, 1950–1959, 1960–1969, ≥ 1970) and adjusted for study site, race/ethnicity, body mass index, education, and hormonal birth control use

^b^Model 2 = Interaction models that include an interaction term with FRP as estimated by 1-year BOADICEA risk score

^c^Smokers are defined as having smoked at least one cigarette per day for 3 months or longer

^d^Regular drinkers are defined as consuming one alcoholic beverage at least once a week for 6 months or longer

The overall findings for smoking and alcohol consumption were similar in sensitivity analyses when limiting to only confirmed invasive BCs and excluding those with diagnosis of (non-breast) cancer prior to baseline (results not shown). The results in Table 2, 3, and 4 by ER status were also similar when we used a competing risk framework for ER censoring (results not shown). We did not observe significant associations with BC risk for smoking intensity, age at smoking initiation, and smoking duration, and there was no evidence of multiplicative interaction between any measure of FRP and any of these smoking and alcohol variables (Additional file 1: Table S1). The overall findings for smoking and alcohol were similar in models excluding *BRCA1* and *BRCA2* mutation carriers (Additional file 1: Table S2).

## Discussion

We did not observe a statistically significant increase in BC risk associated with alcohol or tobacco consumption when considering the cohort as a whole, but we did observe some differences by FRP and ER status. Specifically, alcohol intake was associated with increased risk of ER-positive BC for women at lower predicted absolute risk. For women with a high FRP (above 95th percentile or a 5-year BOADICEA of 6.55%) who also consumed alcohol, smoking was associated with increased overall BC risk.

Five previous studies have reported increased BC risk associated with smoking only for women with a family history of BC or in *BRCA1* and *BRCA2* mutation carriers [32–36]. This includes a report from the UK Generations study cohort of a 35% increased BC risk (HR 1.35; 95 CI 1.12–1.62) for women with a family history of BC who ever smoked, and no increased risk for smokers with no family history of BC [32]. Similarly, the Minnesota breast cancer family study reported ever smoking was associated with a 2.4-fold increased risk for daughters or sisters of women with BC, but not for their nieces or granddaughters [34]. A secondary analysis of high-risk women enrolled in the National Surgical Adjuvant Breast and Bowel Project Breast Cancer Prevention Trial reported that smoking has a greater influence on BC risk for women with an elevated risk of BC [35]. Additionally, we previously reported an association between smoking and increased BC risk for *BRCA1* and *BRCA2* mutation carriers aged less than 50 years [36]. In the current study, we found a positive interaction between smoking and FRP only for regular alcohol drinkers, pointing to a possible synergistic relationship between FRP, smoking, and alcohol with respect to BC risk. This is consistent with three previous studies, including a reanalysis of over 22,000 BC cases and a pooled analysis of 14 cohort studies that examined the smoking association by alcohol consumption and found significant associations with measures of smoking only for alcohol drinkers [8, 19, 32].

We observed evidence for a negative interaction between FRP and alcohol intake in association with ER-positive disease. As previously reported, our family-based cohort is comprised of women across a large range of familial risk [12]. In addition to being enriched with women at higher than average risk, over 30% of cohort participants are at general population risk (5-year BOADICEA < 1.67%), similar to other cohorts unselected for underlying risk. As such, our finding of an increased risk for higher alcohol intake in women at the lower end of the FPR spectrum, which translates into a 5-year BOADICEA < 1.25%, is consistent with previous reports from average-risk populations of a stronger association between alcohol intake and risk of hormone receptor positive tumors [5].

We were limited to active smoking exposure and did not have information on exposure to environmental tobacco smoke (ETS). Some studies have reported an association with ETS and elevated BC risk, as reviewed elsewhere [37]. Another limitation is that information on smoking and alcohol came from self-report of recalled information, which may not be accurate, particularly with respect to amount, frequency, and duration of alcohol intake. However, because the present study was prospective, any measurement error would be non-differential. We also did not have information on binge drinking and could not assess this association with BC risk. Additionally, the prevalence of reported alcohol consumption in our population was low, so we had limited power to fully assess interaction with FRP at different levels of alcohol consumption, particularly for heavier drinkers and by BC subtype. Similarly, for cigarette smoking, our study was limited by a small number of cases who smoked to fully assess interaction with FRP, particularly by BC subtype.

Our study is strengthened by the comprehensive definition of family history that incorporates pedigree information and age at diagnosis of the relatives, extending beyond the conventional binary variable to cover the entire familial risk profile. The heterogeneity of the cohort with respect to family history allowed us to evaluate women across the full spectrum of risk and, in particular, women at high familial risk.

## Conclusion

Findings from this large prospective cohort including high-risk women indicate that there is an elevated risk of ER-positive BC associated with alcohol consumption for women at average population risk (5-year BOADICEA < 1.25%). Additionally, while smoking is not strongly associated with BC, and this association is not modified by underlying FRP, there is an increased overall BC risk for women at very high familial risk (BOADICEA > 6.55%) who also regularly consumed alcohol. These findings can have implications in terms of absolute risk reduction, as alcohol and smoking are modifiable risk factors and present risk reduction opportunities for women across the spectrum of familial risk.

## Supplementary information

**Additional file 1: Table S1.** Adjusted hazard ratios and 95% confidence intervals from Cox proportional hazards modeling of smoking and alcohol variables. **Table S2.** Adjusted hazard ratios and 95% confidence intervals from Cox proportional hazards modeling of smoking and alcohol variables, excluding *BRCA1* and *BRCA2* mutation carriers.

## Data Availability

The datasets analyzed during the current study are available from the corresponding authors on reasonable request.

## Abbreviations

BC: Breast cancer
BCFR: Breast Cancer Family Registry
BOADICEA: Breast Ovarian Analysis of Disease Incidence and Carrier Estimation Algorithm
CI: Confidence interval
ER: Estrogen receptor
FRP: Familial risk profile
HR: Hazard ratio
kConFab: Kathleen Cuningham Foundation Consortium for research into Familial Breast cancer
ProF-SC: Prospective Family Study Cohort
