# Supplementary material for: Alcohol consumption, cigarette smoking, and familial breast cancer risk: findings from the Prospective Family Study Cohort (ProF-SC)
Source: Breast Cancer Res. 2019 Nov 28;21:128. doi: 10.1186/s13058-019-1213-1 (PMC6883541; doi:10.1186/s13058-019-1213-1)
Supplement: Supplementary file 1 — Additional file 1: Table S1. Adjusted hazard ratios and 95% confidence intervals from Cox proportional hazards modeling of smoking and alcohol variables. Table S2. Adjusted hazard ratios and 95% confidence intervals from Cox proportional hazards modeling of smoking and alcohol variables, excluding BRCA1 and BRCA2 mutation carriers. [file 13058_2019_1213_MOESM1_ESM.docx]

# Table S1: Adjusted hazard ratios and 95% confidence intervals from Cox proportional hazards modeling of smoking and alcohol variables

|  | Person Years | BCs | Model 1 ^a^ | Model 2 ^b^ |
| --- | --- | --- | --- | --- |
|  |  |  | HR (95% CI) | HR (95% CI) |
| **Age at Drinking Initiation (years) ^d^** |  |  |  |  |
| Non-regular drinker ^c^ | 85,910.7 | 471 | **Reference** | **Reference** |
| < 18 | 12,617.2 | 73 | 1.20 (0.92, 1.55) | 1.13 (0.84, 1.51) |
| 18 - <21 | 32,677.7 | 183 | 1.08 (0.89, 1.30) | 1.08 (0.87, 1.34) |
| 21 - <30 | 22,451.2 | 131 | 0.97 (0.79, 1.19) | 1.01 (0.80, 1.27) |
| ≥30 | 18,914.7 | 127 | 0.99 (0.80, 1.21) | 1.17 (0.92, 1.50) |
| < 18 *FRP |  |  |  | 1.09 (0.90, 1.32) |
| 18 - <21 *FRP |  |  |  | 1.01 (0.86, 1.18) |
| 21 - <30 *FRP |  |  |  | 0.96 (0.81, 1.14) |
| ≥30 *FRP |  |  |  | 0.82 (0.69, 0.97) |
| **Drinking duration (years) and number of drinks per week ^e^** |  |  |  |  |
| Non-regular drinker ^c^ | 91,046.2 | 502 | **Reference** | **Reference** |
| Duration < 6 yrs, < 7 drinks/wk | 12,540.1 | 55 | 0.97 (0.72, 1.29) | 0.93 (0.67, 1.30) |
| Duration < 6 yrs, ≥ 7 drinks/wk | 5,500.7 | 17 | 0.73 (0.45, 1.20) | 0.79 (0.47, 1.32) |
| Duration 6 - <15 yrs, < 7 drinks/wk | 13,607.3 | 58 | 0.85 (0.64, 1.13) | 0.74 (0.54, 1.03) |
| Duration 6 - <15 yrs, ≥ 7 drinks/wk | 7,935.2 | 44 | 1.12 (0.80, 1.55) | 1.03 (0.71, 1.50) |
| Duration 15 - <25 yrs, < 7 drinks/wk | 11,956.1 | 84 | 1.13 (0.89, 1.44) | 1.19 (0.89, 1.58) |
| Duration 15 - <25 yrs, ≥7 drinks/wk | 8,051.0 | 66 | 1.35 (1.03, 1.77) | 1.35 (0.98, 1.85) |
| Duration ≥ 25 yrs, < 7 drinks/wk | 10,003.6 | 74 | 1.06 (0.82, 1.37) | 1.23 (0.89, 1.70) |
| Duration ≥ 25 yrs, ≥ 7 drinks/wk | 8,324.5 | 67 | 1.11 (0.85, 1.46) | 1.59 (1.15, 2.21) |
| Duration < 6 yrs, < 7 drinks/wk * FRP |  |  |  | 1.06 (0.82, 1.37) |
| Duration < 6 yrs, ≥ 7 drinks/wk *FRP |  |  |  | 0.90 (0.64, 1.25) |
| Duration 6 - <15 yrs, < 7 drinks/wk *FRP |  |  |  | 1.20 (0.97, 1.49) |
| Duration 6 - <15 yrs, ≥ 7 drinks/wk *FRP |  |  |  | 1.12 (0.88, 1.43) |
| Duration 15 - <25 yrs, < 7 drinks/wk *FRP |  |  |  | 0.94 (0.74, 1.18) |
| Duration 15 - <25 yrs, ≥7 drinks/wk *FRP |  |  |  | 1.01 (0.77, 1.32) |
| Duration ≥ 25 yrs, < 7 drinks/wk *FRP |  |  |  | 0.84 (0.64, 1.09) |
| Duration ≥ 25 yrs, ≥ 7 drinks/wk *FRP |  |  |  | 0.65 (0.49, 0.85) |
| **Smoking Intensity: cigarettes smoked per day (cig/day) ^g^** |  |  |  |  |
| Never smoker ^f^ | 102,618.8 | 559 | **Reference** | **Reference** |
| 1 - <5 cig/day | 11,972.6 | 76 | 1.13 (0.88, 1.44) | 1.30 (0.98, 1.72) |
| 5 - <10 cig/day | 13,205.7 | 63 | 0.92 (0.71, 1.20) | 0.88 (0.64, 1.21) |
| 10 - <20 cig/day | 22,660.4 | 128 | 1.00 (0.83, 1.22) | 0.94 (0.75, 1.18) |
| ≥ 20 cig/day | 25,089.0 | 169 | 1.11 (0.93, 1.32) | 1.17 (0.95, 1.43) |
| 1 - <5 cig/day * FRP |  |  |  | 0.83 (0.66, 1.04) |
| 5 - <10 cig/day *FRP |  |  |  | 1.10 (0.80, 1.51) |
| 10 - <20 cig/day *FRP |  |  |  | 1.08 (0.94, 1.25) |
| ≥ 20 cig/day *FRP |  |  |  | 0.93 (0.81, 1.08) |
| **Age at Smoking Initiation (years) ^g^** |  |  |  |  |
| Never smoker ^f^ | 102,618.8 | 559 | **Reference** | **Reference** |
| < 16 | 17,256.7 | 95 | 1.05 (0.84, 1.32) | 1.18 (0.83, 1.66) |
| 16 - <18 | 19,812.2 | 117 | 1.09 (0.89, 1.33) | 1.03 (0.73, 1.44) |
| 18 - <20 | 18,543.9 | 116 | 1.04 (0.85, 1.27) | 1.00 (0.65, 1.54) |
| ≥ 20 | 17,611.3 | 111 | 1.03 (0.84, 1.27) | 0.97 (0.67, 1.40) |
| < 16 *FRP |  |  |  | 0.87 (0.49, 1.53) |
| 16 - <18 *FRP |  |  |  | 1.08 (0.61, 1.91) |
| 18 - <20 *FRP |  |  |  | 1.17 (0.53, 2.59) |
| ≥ 20 *FRP |  |  |  | 1.05 (0.58, 1.90) |
| **Smoking Duration (years)^h^** |  |  |  |  |
| Never smoker ^f^ | 102,618.8 | 571 | **Reference** | **Reference** |
| <10 | 24,638.7 | 114 | 0.90 (0.73, 1.10) | 0.94 (0.75, 1.18) |
| 10 - <20 | 20,318.6 | 143 | 1.27 (1.05, 1.53) | 1.17 (0.94, 1.46) |
| 20 - <30 | 13,099.8 | 93 | 1.10 (0.88, 1.38) | 1.15 (0.88, 1.50) |
| ≥ 30 | 12,265.7 | 87 | 1.02 (0.81, 1.29) | 1.19 (0.89, 1.59) |
| <10 *FRP |  |  |  | 0.93 (0.79, 1.11) |
| 10 - <20 *FRP |  |  |  | 1.11 (0.97, 1.28) |
| 20 - <30 *FRP |  |  |  | 0.95 (0.79, 1.14) |
| ≥ 30 *FRP |  |  |  | 0.84 (0.66, 1.07) |
| *BCs = breast cancers, HR = hazard ratio, CI = confidence interval , ER = estrogen hormone receptor expression, FRP = familial risk profile* | | | | |
| ^a^ Model 1= Adjusted Cox models which are stratified by birth cohort (<1950, 1950-1959, 1960-1969, ≥1970) and adjusted for study center, race/ethnicity, and FRP as estimated by 1-year BOADICEA risk score. | | | | |
| ^b^ Model 2= Interaction models that include an interaction term with FRP as estimated by 1-year BOADICEA risk score. | | | | |
| ^c^ Regular drinkers are defined as consuming one alcoholic beverage at least once a week for 6 months or longer. | | | | |
| ^d^ Model is also adjusted for BMI, education, parity/breastfeeding, hormonal birth control use, age at menarche, and cigarette smoking (current, former, never). | | | | |
| ^e^ Model is also adjusted for BMI and education. | | | | |
| ^f^ Smokers are defined as having smoked at least one cigarette per day for 3 months or longer. | | | | |
| ^g^ Model is also adjusted for BMI, education, and hormonal birth control use. | | | | |
| ^h^ Model is also adjusted for education. | | | | |

# Table S2: Adjusted hazard ratios and 95% confidence intervals from Cox proportional hazards modeling of smoking and alcohol variables, excluding *BRCA1* and *BRCA2* mutation carriers

|  | Person Years | BC | Model 1 ^a^ | Model 2 ^b^ |
| --- | --- | --- | --- | --- |
|  |  |  | HR (95% CI) | HR (95% CI) |
| **Smoking Status** |  |  |  |  |
| Never smoker ^c^ | 96,768.7 | 450 | **Reference** | **Reference** |
| Former Smoker | 45,250.6 | 262 | 1.10 (0.94, 1.28) | 1.11 (0.86, 1.43) |
| Current Smoker | 25,216.2 | 104 | 0.96 (0.78, 1.19) | 0.90 (0.64, 1.26) |
| Former Smoker*FRP |  |  |  | 0.97 (0.64, 1.48) |
| Current Smoker*FRP |  |  |  | 1.17 (0.64, 2.14) |
| **Alcoholic drinks/week** |  |  |  |  |
| Non-regular drinker ^d^ | 84,655.3 | 385 | **Reference** | **Reference** |
| < 7 drinks/week | 46,745.2 | 230 | 1.06 (0.89, 1.26) | 1.04 (0.78, 1.38) |
| ≥ 7 drinks/week | 28,560.8 | 166 | 1.21 (0.99, 1.47) | 1.28 (0.97, 1.69) |
| < 7 drinks/week*FRP |  |  |  | 1.04 (0.64, 1.68) |
| ≥ 7 drinks/week*FRP |  |  |  | 0.88 (0.58, 1.34) |
| **Smoking status by categories of drinking** |  |  |  |  |
| ***Regular drinkers ^d^*** |  |  |  |  |
| Never smoker | 38,516.1 | 188 | **Reference** | **Reference** |
| Former Smoker | 30,611.0 | 194 | 1.18 (0.96, 1.44) | 1.15 (0.83, 1.57) |
| Current Smoker | 15,892.3 | 65 | 0.91 (0.69, 1.20) | 0.76 (0.50, 1.17) |
| Former Smoker*FRP |  |  |  | 1.06 (0.62, 1.80) |
| Current Smoker*FRP |  |  |  | 1.52 (0.72, 3.21) |
| ***Non-regular drinkers*** |  |  |  |  |
| Never smoker | 58,089.9 | 262 | **Reference** | **Reference** |
| Former Smoker | 14,539.6 | 68 | 0.89 (0.68, 1.16) | 0.90 (0.59, 1.38) |
| Current Smoker | 9,292.4 | 39 | 1.04 (0.74, 1.45) | 1.12 (0.64, 1.94) |
| Former Smoker*FRP |  |  |  | 0.97 (0.49, 1.91) |
| Current Smoker*FRP |  |  |  | 0.84 (0.30, 2.33) |
| *BC = breast cancer, HR = hazard ratio, CI = confidence interval , FRP = familial risk profile* | | | |  |
| ^a^ Model 1= Adjusted Cox models which are stratified by birth cohort (<1950, 1950-1959, 1960-1969, ≥1970) and adjusted for study site, race/ethnicity, BMI, education, hormonal birth control use and FRP as estimated by 1-year BOADICEA risk score. Alcoholic drinks per week models also control for cigarette smoking (current, former, never). | | | | |
| ^b^ Model 2= Interaction models that include an interaction term with FRP as estimated by 1-year BOADICEA risk score. | | | | |
| ^c^ Smokers are defined as having smoked at least once cigarette per day for 3 months or longer. | | | | |
| ^d^ Regular drinkers are defined as consuming one alcoholic beverage at least once a week for 6 months or longer. | | | | |
